# Association Between Sedative-Hypnotic Medication Use and Fall-Related Emergency Department Visits Among Older Adults

**DOI:** 10.7759/cureus.107830

**Published:** 2026-04-27

**Authors:** Akinyele Oladimeji, Kwasi A Opoku, Angel Dockery, Dana Alcin, Victor C Ofochukwu, Azeberoje Osueni, Emeka K Okobi

**Affiliations:** 1 Family Medicine, Alberta Health Services, Edmonton, CAN; 2 Internal Medicine, St. Theresa's Hospital, Nkoranza, GHA; 3 Anesthesiology, University of the West Indies, Mona, Kingston, JAM; 4 Medicine, University of Houston College of Medicine, Houston, USA; 5 Medicine, Ebonyi State University, Abakaliki, NGA; 6 Medicine and Surgery, Hospital Corporation of America (HCA) Houston Healthcare Pearland, Pearland, USA; 7 Gastroenterology and Hepatology, Lagos University Teaching Hospital, Lagos, NGA; 8 Maxillofacial Surgery, Ahmadu Bello University Teaching Hospital, Zaria, NGA

**Keywords:** emergency department, falls, nhamcs, older adults, polypharmacy, sedative hypnotics

## Abstract

Background: Falls are a leading cause of injury and emergency department visits among older adults. Medication use, including sedative hypnotics, may influence fall risk, but findings remain inconsistent across clinical settings.

Objective: This study examined the association between sedative hypnotic use and fall-related emergency department visits among older adults.

Methods: A retrospective cross-sectional analysis was conducted using the National Hospital Ambulatory Medical Care Survey emergency department data from 2014 to 2020. The study included adults aged 65 years and older. Fall-related visits were identified using external cause-of-injury codes, and sedative use was defined using Multum therapeutic class codes. Survey-weighted descriptive analyses were performed, and group comparisons were assessed using survey-adjusted t-tests and chi-square tests. Multivariable, survey-weighted logistic regression was used to evaluate associations while adjusting for demographic and clinical factors.

Results: The analytic sample included 21,227 visits, representing 155,790,536 weighted visits. Fall-related visits were more common among older individuals and those with dementia. Sedative use was associated with lower odds of fall-related visits after adjustment for covariates (adjusted odds ratio = 0.74; 95% confidence interval = 0.55-0.98; p = 0.034). Age and dementia were associated with higher odds, while male sex and non-Hispanic Black, Hispanic, and non-Hispanic other groups had lower odds compared with non-Hispanic White individuals.

Conclusion: Sedative use was associated with lower odds of fall-related emergency visits in this population. These findings should be interpreted in the context of potential residual confounding and highlight the need for further research using longitudinal data.

## Introduction

According to the World Health Organization, a fall is defined as an incident that compels an individual to lie down unintentionally on the floor, ground, or other lower levels, and is associated with high morbidity and mortality [1]. Worldwide, it is estimated that between 28% and 35% of persons aged 65 and older fall each year, and the prevalence rate also increases with age [2,3]. The falls have been proven to cause severe negative results that include injury, hospitalization, long-term care, fear of falls, activity limitation, social isolation, reduced self-efficacy and quality of life, and even mortality [4]. Both falls and fear of falls may significantly decrease quality of life and independence, and hence lead to institutionalization in an elderly individual [5]. Among older adults who dwell in the community, major modifiable risks are gait and balance disorders, orthostatic hypotension, sensory impairment, medications, and hazards of the environment [6].

The primary benefits of nonbenzodiazepine sedative hypnotics over benzodiazepines in older adults are a reduced half-life, improved sleep architecture, and reduced rebound and withdrawal, which might also explain fewer falls than older benzodiazepine agents [7]. Sedative hypnotic therapy is the most frequently used in the treatment of insomnia, which lowers the number of falls in the elderly [8]. Sedative-hypnotic drugs, in particular, benzodiazepines and Z-drugs, have been suggested as potentially causing falls and fractures [9]. Older sedative hypnotics like triazolam and even newer sedatives like zolpidem have been reported to cause loss of memory and confusion in patients of all ages, where the chances of the same might be very high in patients of old age [10]. Though drug therapy has played a major role in the management of various medical conditions among older patients, a significant number of these patients will be experiencing some form of adverse drug reaction [11].

Earlier studies have proposed that there is a correlation between the use of sedative medication and the risk of falls, but the evidence has proved inconclusive, and the degree of association is not well defined [12,13]. According to some studies, there is a close relationship between fall-related injuries and the use of sedatives, and there are studies that suggest that the risk might differ based on the dosage, duration of use, and the environmental factors affecting an individual patient [14]. Moreover, evidence examining the association between sedative-hypnotic use and fall-related emergency department visits remains limited. Fall-related emergency visits represent clinically distinct outcomes compared with general fall risk or fall-related injuries, as they capture events requiring acute medical care, particularly in the presence of symptoms such as confusion or altered mental status [15,16]. This knowledge gap highlights the need for further research on the association between sedative-hypnotic use and fall-related outcomes, particularly those resulting in emergency department visits among older adults [17].

Emergency department visits related to falls are clinically significant events that often reflect injury severity, healthcare utilization, and underlying risk factors that require urgent care. Focusing on fall-related emergency visits provides a more clinically relevant perspective compared with general fall risk, as these encounters are more likely to capture serious outcomes and healthcare burden [18]. Understanding the association between sedative-hypnotic use and fall-related emergency department visits may provide useful context for clinicians and support ongoing evaluation of prescribing practices and patient care approaches in older adults [19]. In addition, risk population identification could be used to facilitate specific interventions that minimize medication-related harm and enhance overall patient outcomes [20].

The objective of this study is to assess the association between sedative medication use and fall-related emergency department visits among adults aged ≥65 years. Importantly, the findings of this study will provide insights to guide practitioners in minimizing adverse events and maximizing pharmacological results.

## Materials and methods

Study design and data source

This study used a retrospective cross-sectional design based on data from the National Hospital Ambulatory Medical Care Survey (NHAMCS) emergency department component for the years 2014 through 2020 [21]. Given the cross-sectional nature of the data, the temporal relationship between sedative use and fall-related emergency department visits cannot be established, and the findings should be interpreted as associations. Additionally, the possibility of reverse causation cannot be excluded. The survey is conducted by the National Center for Health Statistics and uses a multistage probability sampling design to produce nationally representative estimates of emergency department visits in the United States. Data for NHAMCS are collected by trained hospital staff using standardized data collection forms and are abstracted from patient medical records, including documentation by physicians and clinical personnel. The sampling design incorporates stratification, clustering, and weighting to account for unequal probabilities of selection. Publicly available data files for each year were combined into a single analytic dataset, and a survey year variable was created to identify the source year of each observation.

Study population

The study population included emergency department visits among adults aged 65 years or older. Because NHAMCS is a visit-level dataset, individual patients cannot be uniquely identified, and multiple visits from the same individual may be included. As a result, observations may not be fully independent, which could influence the estimated associations. Visits with missing or invalid responses for the injury variable were excluded, including responses coded as unknown or blank. These exclusions were applied to ensure accurate classification of fall-related visits. However, if missingness is associated with exposure or outcome status, this approach may introduce selection bias, which should be considered when interpreting the results. Visits classified as questionable injury status were also excluded to ensure a clear definition of injury-related encounters. The final analytic sample consisted of 21,227 unweighted visits, representing 155,790,536 weighted emergency department visits nationally. Applying these exclusion criteria removed some observations from the initial dataset. Detailed characterization of excluded visits was limited; however, if excluded observations differ systematically from those included (e.g., by age, sex, or other factors), this may affect the representativeness of the final sample.

Variables and measures

The primary outcome was a fall-related emergency department visit. This variable was defined using external cause of injury codes, where visits with codes corresponding to falls within the range W00-W19 were classified as fall-related, and were further restricted to visits identified as injury-related. Visits with "questionable injury status" were excluded; this category included records with unclear, inconsistent, or nonspecific injury coding that could not be reliably classified as injury-related or noninjury-related based on available variables. While external cause codes are commonly used to identify fall-related visits in administrative datasets, their sensitivity may be limited, particularly when falls are not the primary reason for the visit. The primary exposure was sedative hypnotic medication use, defined using therapeutic class codes from the Multum Lexicon. Medications classified as sedative or hypnotic agents were identified using Multum therapeutic class codes 069, 070, and 071, corresponding to anxiolytics, sedatives, and hypnotics. These categories include commonly used agents such as benzodiazepines (e.g., lorazepam and diazepam), nonbenzodiazepine hypnotics (e.g., zolpidem, eszopiclone, and zaleplon), and other sedative agents captured within these classes. Medication data in NHAMCS reflect drugs documented at the time of the emergency department visit and may include both home medications and those administered or prescribed during the visit. As such, the timing of medication use relative to the fall-related event cannot be determined. These codes were assessed for all recorded medications at each visit, and a binary variable was created to indicate the presence of any sedative-hypnotic medication. This binary classification does not capture differences in dosage, duration, or specific drug classes, which may have varying risk profiles. As a result, exposure may be misclassified and may attenuate observed associations. Covariates included demographic and clinical characteristics selected a priori based on clinical relevance and data availability. These included age as a continuous variable, sex, and race and ethnicity categorized as non-Hispanic White, non-Hispanic Black, Hispanic, and non-Hispanic other. Clinical comorbidities included dementia, stroke, diabetes, and depression, each defined as a binary indicator based on survey variables. Hypertension was not included due to limitations in consistent variable availability and completeness across the study sample. Within NHAMCS, polypharmacy was defined as the use of five or more medications during the visit. Certain potentially relevant confounders, including vision impairment, gait or balance disorders, and environmental risk factors, are not available in NHAMCS and therefore could not be included in the analysis.

Missing data

A complete-case analysis approach was used after excluding observations with missing or invalid injury-related data. Specifically, 1,147 visits with missing or invalid injury responses and 179 visits with questionable injury classification were excluded, resulting in the final analytic sample of 21,227 visits. The final dataset, therefore, contained no missing values for variables included in the analysis. However, this approach assumes that missingness does not differ systematically by exposure or outcome; if this assumption is not met, the exclusions may introduce selection bias and affect the representativeness of the sample.

Statistical analysis

All analyses accounted for the complex survey design using the provided patient visit weights, primary sampling units, and strata variables to produce nationally representative estimates. Descriptive statistics were used to summarize patient characteristics by fall-related visit status. Continuous variables were described using means and standard deviations, and categorical variables were summarized using weighted counts and row percentages. Group comparisons by the outcome variable were conducted using survey-adjusted t-tests for continuous variables and survey-adjusted chi-square tests for categorical variables. Multivariable survey-weighted logistic regression was used to examine the association between sedative hypnotic use and fall-related visits, adjusting for age, sex, race and ethnicity, dementia, stroke, diabetes, depression, and polypharmacy. Results were reported as adjusted odds ratios (ORs) with 95% confidence intervals (CIs). Multicollinearity was assessed using variance inflation factors (VIFs), which ranged from 1.01 to 1.09, with a mean VIF of 1.04, indicating no evidence of multicollinearity. All analyses were conducted in Stata version 18 (StataCorp LLC, College Station, TX) [22].

Ethical considerations

The study used publicly available deidentified data and did not involve direct interaction with human participants. As such, it was exempt from institutional review board approval according to standard guidelines for research using secondary data without identifiable information.

## Results

Table 1 presents the baseline characteristics of older adults by fall-related emergency department visit status.

**Table 1:** Baseline characteristics of older adults by fall-related ED visit (NHAMCS 2014-2020; unweighted n = 21,227; weighted n = 155,790,536) Values are weighted national estimates from NHAMCS. Continuous variables are presented as weighted mean ± SD. Categorical variables are presented as weighted counts (N) with corresponding column percentages (%) in the format n (%). Percentages represent column proportions. Group comparisons were performed using survey-adjusted t-tests for continuous variables and survey-adjusted chi-square tests for categorical variables. p values are presented for descriptive purposes only. Given the large sample size, statistical significance may not reflect clinically meaningful differences; therefore, interpretation is based on the magnitude and pattern of differences rather than p values alone. All analyses accounted for sampling weights, strata, and clustering ^*^Statistical significance was set at p < 0.05 SD: standard deviation; ED: emergency department; NHAMCS: National Hospital Ambulatory Medical Care Survey Source: The table was generated by the authors using Stata version 18 [22]

Variable	No fall (n = 137,423,121)	Fall-related visit (n = 18,367,415)	Test statistic	p value
Age, years, mean (SD)	76.29 (8.12)	78.62 (8.41)	t = 8.90	<0.001^*^
Sedative use, n (%)				
No sedative	126,396,676 (92.0%)	17,382,437 (94.7%)	F = 9.56	0.002^*^
Sedative use	11,026,445 (8.0%)	984,978 (5.3%)		
Gender, n (%)				
Female	76,544,445 (55.7%)	11,810,501 (64.3%)	F = 28.04	<0.001^*^
Male	60,878,676 (44.3%)	6,556,914 (35.7%)		
Race/ethnicity, n (%)				
Non-Hispanic White	101,028,590 (73.5%)	15,257,758 (83.1%)	F = 19.25	<0.001^*^
Non-Hispanic Black	19,210,384 (14.0%)	1,618,336 (8.8%)		
Hispanic	12,363,468 (9.0%)	1,035,070 (5.6%)		
Non-Hispanic other	4,820,679 (3.5%)	456,251 (2.5%)		
Dementia, n (%)				
No	128,736,152 (93.7%)	16,279,815 (88.6%)	F = 42.84	<0.001^*^
Yes	8,686,969 (6.3%)	2,087,600 (11.4%)		
Stroke, n (%)				
No	122,003,050 (88.8%)	15,840,676 (86.3%)	F = 6.52	0.011^*^
Yes	15,420,071 (11.2%)	2,526,739 (13.7%)		
Diabetes, n (%)				
No	115,630,781 (84.2%)	16,021,662 (87.2%)	F = 8.50	0.004^*^
Yes	21,792,340 (15.8%)	2,345,753 (12.8%)		
Depression, n (%)				
No	121,617,650 (88.5%)	15,921,022 (86.7%)	F = 3.88	0.049^*^
Yes	15,805,471 (11.5%)	2,446,393 (13.3%)		
Polypharmacy, n (%)				
No	107,524,971 (78.2%)	15,399,653 (83.8%)	F = 15.26	<0.001^*^
Yes	29,898,150 (21.8%)	2,967,762 (16.2%)		

The results indicate that the mean age was higher among fall-related visits than among nonfall visits, 78.62 (8.41) vs. 76.29 (8.12), with a statistically significant difference based on the survey-adjusted t-test (p < 0.001). Sedative use differed between the groups, where visits with no sedative exposure included 126,396,676 (92.0%) nonfall visits and 17,382,437 (94.7%) fall-related visits, while visits with sedative use included 11,026,445 (8.0%) nonfall visits and 984,978(5.3%) fall-related visits, with a significant difference based on the survey-adjusted chi-square test (p = 0.002). Gender distribution also differed, with women accounting for 76,544,445 (55.7%) nonfall visits and 11,810,501 (64.3%) fall-related visits, and men accounting for 60,878,676 (44.3%) nonfall visits and 6,556,914 (35.7%) fall-related visits (p < 0.001). Differences across race and ethnicity were observed, with non-Hispanic White visits showing 101,028,590 (73.5%) nonfall and 15,257,758 (83.1%) fall-related visits, while non-Hispanic Black, Hispanic, and non-Hispanic other groups showed lower proportions of fall-related visits (p < 0.001).

**Figure 1:**
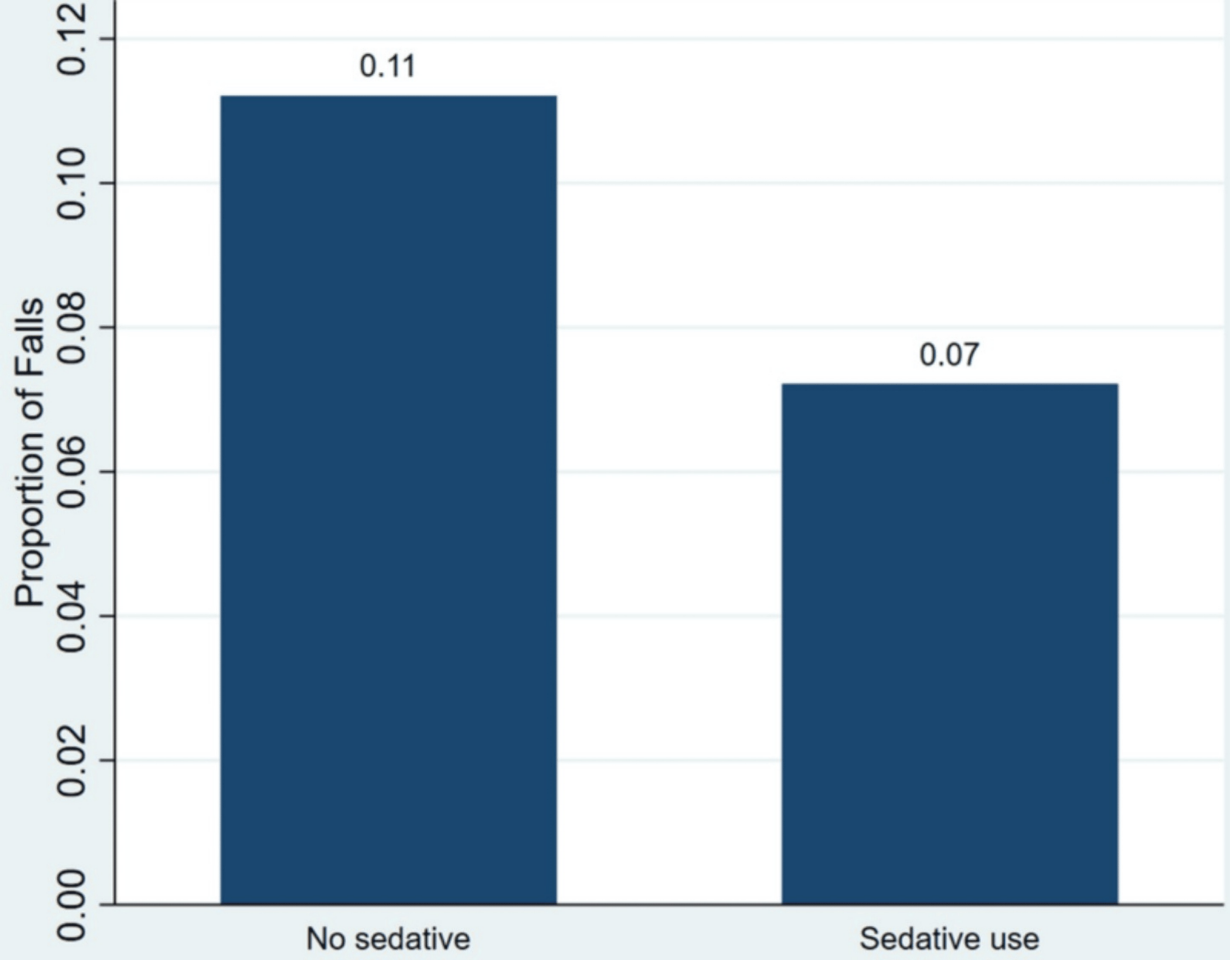
Proportion of fall-related ED visits by sedative use ED: emergency department

Clinical characteristics showed variation by fall status. Dementia was more frequent among fall-related visits, with 8,686,969 (6.3%) nonfall and 2,087,600 (11.4%) fall-related visits among those with dementia, compared with 128,736,152 (93.7%) and 16,279,815 (88.6%) among those without dementia (p < 0.001). Stroke also differed between the groups, with higher proportions of fall-related visits among those with stroke (p = 0.011). Diabetes showed a smaller difference, with 115,630,781 (84.2%) nonfall and 16,021,662 (87.2%) fall-related visits among those without diabetes, compared with 21,792,340 (15.8%) and 2,345,753 (10.0%) among those with diabetes (p = 0.004). Depression was slightly more common among fall-related visits (p = 0.049). Polypharmacy differed between the groups, with 107,524,971 (78.2%) nonfall and 15,399,653 (83.8%) fall-related visits among those without polypharmacy, compared with 29,898,150 (21.8%) and 2,967,762 (16.2%) among those with polypharmacy (p < 0.001). Figure 1 illustrates the proportion of fall-related emergency department visits by sedative use.

Figure 1 shows that the proportion of fall-related visits was higher among visits without sedative use compared with those with sedative use. The difference between the groups is visually apparent, with nonsedative visits showing a higher proportion of falls than sedative visits. Table 2 presents the multivariable survey-weighted logistic regression analysis of factors associated with fall-related emergency department visits.

**Table 2:** Multivariable survey-weighted logistic regression of factors associated with fall-related ED visits (NHAMCS 2014-2020) Estimates are derived from survey-weighted logistic regression accounting for strata, clustering, and sampling weights. All variables listed were included in the adjusted model OR: odds ratio; CI: confidence interval; ED: emergency department; NHAMCS: National Hospital Ambulatory Medical Care Survey Source: The table was generated by the authors using Stata version 18 [22]

Variable	Adjusted OR	95% CI	p value
Sedative use			
Sedative use vs. no sedative	0.74	0.55-0.98	0.034
Age (years)	1.03	1.02-1.03	<0.001
Sex			
Male vs. female	0.72	0.63-0.83	<0.001
Race/ethnicity			
Non-Hispanic Black vs. non-Hispanic White	0.60	0.49-0.73	<0.001
Hispanic vs. non-Hispanic White	0.59	0.46-0.75	<0.001
Non-Hispanic other vs. non-Hispanic White	0.65	0.47-0.90	0.009
Dementia (yes vs. no)	1.53	1.26-1.87	<0.001
Stroke (yes vs. no)	1.20	0.99-1.45	0.064
Diabetes (yes vs. no)	0.85	0.71-1.02	0.079
Depression (yes vs. no)	1.15	0.96-1.37	0.124
Polypharmacy (yes vs. no)	0.74	0.61-0.89	0.002

**Figure 2:**
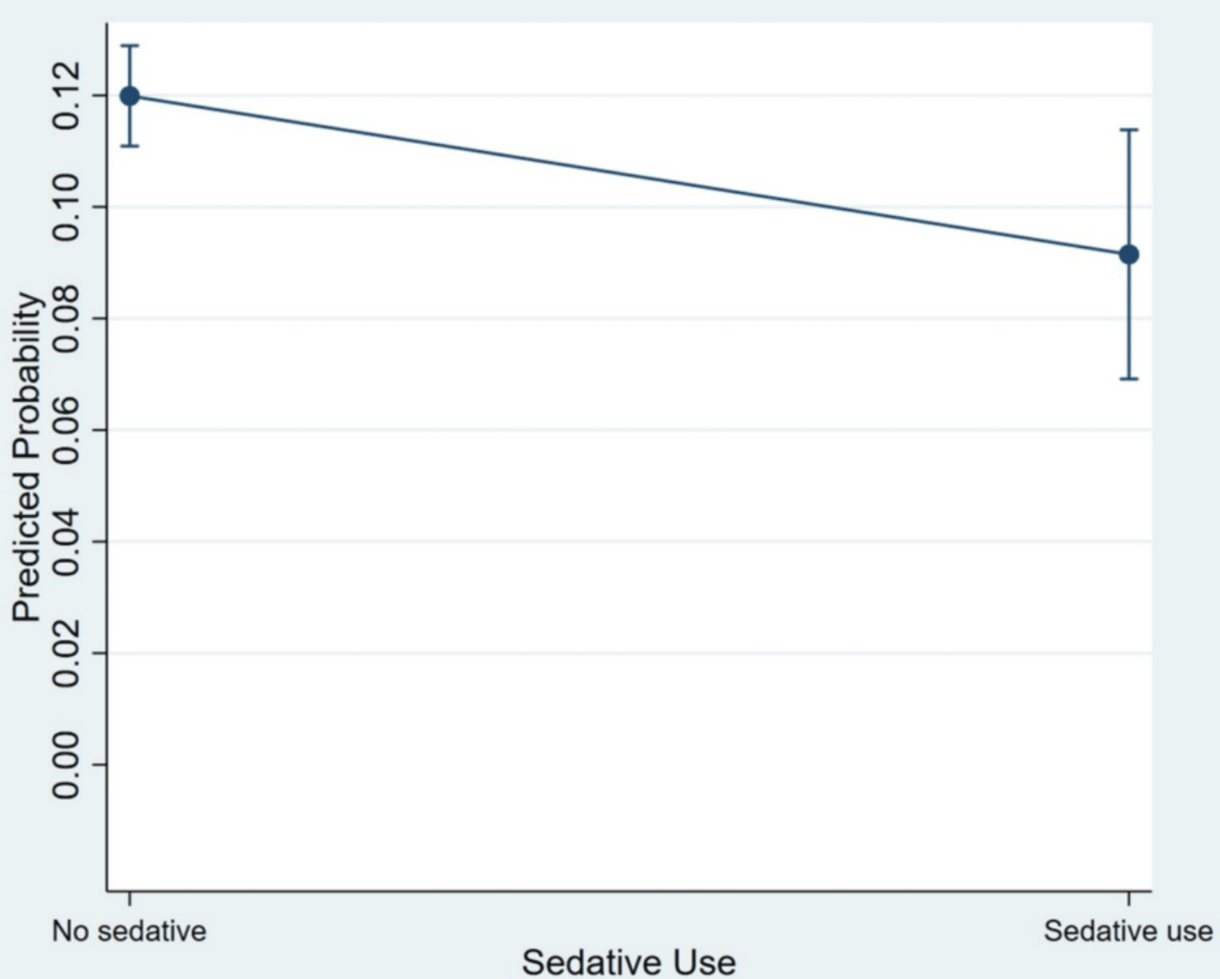
Adjusted predicted probability of fall-related ED visits by sedative use Points represent predicted probabilities derived from survey-weighted logistic regression. Error bars indicate 95% confidence intervals. Predictions are adjusted for age, sex, race and ethnicity, dementia, stroke, diabetes, depression, and polypharmacy ED: emergency department

The results indicate that sedative use was associated with lower odds of fall-related visits compared with no sedative use (adjusted OR = 0.74; 95% CI = 0.55-0.98; p = 0.034). Increasing age was associated with higher odds of fall-related visits (adjusted OR = 1.03 per year; p < 0.001). Male sex was associated with lower odds compared with female sex (adjusted OR = 0.72; p < 0.001). Compared with non-Hispanic White patients, non-Hispanic Black, Hispanic, and non-Hispanic other groups had lower odds of fall-related visits, all statistically significant. Dementia was associated with higher odds of fall-related visits (adjusted OR = 1.53; p < 0.001). Stroke showed a higher odds estimate but was not statistically significant (p = 0.064). Diabetes and depression were not statistically significant predictors in the adjusted model. Polypharmacy was associated with lower odds of fall-related visits (adjusted OR = 0.74; p = 0.002). Figure 2 illustrates the adjusted predicted probability of fall-related visits by sedative use.

Figure 2 shows that the adjusted predicted probability of fall-related visits was higher among visits without sedative use compared with those with sedative use. The CIs do not fully overlap, indicating a statistically significant difference between the two groups after adjustment for covariates.

## Discussion

The present study examined the association between sedative hypnotic use and fall-related emergency department visits among older adults using nationally representative data. The findings show that fall-related visits were more frequent among older individuals, particularly those with dementia and stroke, and that several demographic and clinical factors differed by fall status. Sedative use was associated with a lower likelihood of fall-related visits after adjustment for covariates. Increasing age and dementia were associated with higher odds of falls, while male sex and non-Hispanic Black, Hispanic, and non-Hispanic other groups showed lower odds compared with non-Hispanic White individuals. These findings align with existing evidence that age and comorbid conditions are important contributors to fall risk in older adults [1-3]. Prior studies have consistently shown that cognitive impairment and neurological conditions increase vulnerability to falls through impaired balance, reduced attention, and functional decline [3]. The observed differences by sex and race may reflect variations in health status, care-seeking patterns, or environmental exposures, which have been reported in earlier research on fall-related injuries in emergency settings [18]. The inverse association between sedative use and fall-related visits observed in this study differs from prior literature, which has generally reported an increased risk of falls associated with sedative-hypnotic medications [7,9,12]. This finding should be interpreted with caution and should not be considered evidence of a protective effect. Several methodological factors may explain this result. First, confounding by indication is likely, as patients prescribed sedatives may differ systematically from those not prescribed these medications, including reduced mobility or lower levels of physical activity, which could decrease exposure to fall risk. Second, selective prescribing may occur, whereby clinicians avoid prescribing sedatives to patients perceived to be at high risk for falls, thereby introducing bias into the observed association. Third, survivorship bias may be present, as individuals who tolerate sedative use without adverse events may represent a healthier subset of older adults. In addition, the cross-sectional design precludes assessment of temporal relationships, and medication exposure was measured at the time of the emergency department visit, raising the possibility of reverse causation and exposure misclassification. Furthermore, important confounders such as functional status, frailty, and mobility were not available in the dataset, contributing to residual confounding. Taken together, these limitations indicate that the observed association likely reflects underlying differences in patient characteristics rather than a true protective effect of sedative use. This study cannot determine whether sedative use reduces fall risk, and the findings should be interpreted as associative and hypothesis-generating rather than causal. This contrast may reflect differences in patient selection, medication patterns, or clinical contexts captured in emergency department data. Although the association reached statistical significance, the CI was relatively wide and close to the null, suggesting that the finding should be interpreted with caution.

Clinical guidelines in the United States emphasize the prevention of falls in older adults through risk assessment and careful medication review. Current recommendations highlight the importance of identifying medications that may increase fall risk, including sedative hypnotics, and suggest minimizing their use when possible [6]. Sedative medications are known to affect cognition, coordination, and alertness, and have been associated with increased fall risk in prior studies [10,13]. In contrast, our findings demonstrated an inverse association between sedative use and fall-related emergency department visits. This discrepancy likely reflects methodological limitations rather than a true protective effect. The cross-sectional design does not allow assessment of temporal relationships, and medication exposure was measured at the time of the emergency department visit, raising the possibility of reverse causation. Additionally, exposure misclassification may occur, as recorded medications may include those administered during the visit rather than reflecting chronic use. Residual confounding is also likely, as important factors such as functional status, mobility, and frailty were not available in the dataset. Together, these limitations suggest that the observed inverse association should be interpreted cautiously and may reflect underlying differences in patient characteristics rather than a causal relationship. Glass et al. [10] similarly highlight the risks associated with sedative hypnotics, noting that despite their benefits for insomnia, these medications can increase the likelihood of adverse events such as falls and cognitive impairment in older populations, thus necessitating cautious prescribing and ongoing evaluation.

Moreover, Andrade [13] underscores the need for clinicians to balance the benefits and risks of sedative hypnotic use, recommending regular medication reviews and deprescribing protocols tailored to individual patient risk profiles. This includes comprehensive medication reconciliation at each clinical encounter to identify potentially inappropriate medications and reduce polypharmacy, which has also been linked to fall risk. Routine fall risk assessments, incorporating functional status, cognitive evaluation, and environmental factors, are critical components of current guidelines for identifying high-risk individuals and implementing targeted interventions.

In addition, emergency department data have shown that adverse drug events involving benzodiazepines are common among older adults and may contribute to injury-related visits [15,16]. These findings should be interpreted with caution, as the observed inverse association is likely influenced by methodological limitations, including potential confounding and bias, rather than reflecting a true protective effect of sedative use. As such, the results should not be used to inform clinical prescribing or deprescribing decisions.

Several mechanisms may help explain the associations observed in this study. Advancing age is associated with physiological changes that affect balance, muscle strength, and sensory function, increasing susceptibility to falls [1,2]. Dementia and stroke may further impair mobility and judgment, leading to a higher likelihood of fall-related injuries. Sedative hypnotic medications can alter central nervous system function, which may affect reaction time and postural control [11]. However, the lower odds of falls observed among sedative users in this study may reflect differences in clinical characteristics, such as closer monitoring, reduced activity levels, or selective prescribing practices in higher risk individuals. These patterns may reflect the complex interplay between medication use and patient characteristics rather than a direct effect of sedative medications. Given the limitations of the dataset, including the absence of key variables and its cross-sectional design, this finding should be interpreted as exploratory and hypothesis-generating, and further research with more detailed, longitudinal data is needed to better understand this relationship [20].

Strengths and limitations of the study

This study has several strengths, including the use of nationally representative data and the application of survey methods that account for weighting, clustering, and stratification. The inclusion of multiple clinical and demographic variables allows for adjustment of important confounders. However, several limitations should be considered. The cross-sectional design does not allow assessment of temporal relationships between sedative use and fall-related emergency department visits, and the possibility of reverse causation cannot be excluded. Medication exposure was recorded at the time of the emergency department visit and may include medications administered during care, limiting the ability to distinguish preexisting use and introducing potential misclassification. In addition, the exposure was defined as a binary variable and does not account for differences in medication type, dosage, or duration, which may influence the observed associations. The analysis is based on visit-level data, and repeated visits by the same individual cannot be identified, which may affect the independence of observations. The use of external cause codes to identify fall-related visits may result in under capture, particularly when falls are not the primary reason for the visit. A complete-case approach was used after excluding observations with missing or unclear injury data, which may introduce selection bias if excluded visits differ systematically from those included. Important variables such as functional status, frailty, mobility, environmental hazards, prior fall history, and medication-related factors were not available and could not be included, raising the possibility of residual confounding. Additionally, the grouping of multiple sedative-related medication classes may obscure differences in risk profiles across specific drugs. These limitations should be considered when interpreting the findings. Future research using longitudinal data and more detailed clinical information is needed to better characterize the relationship between sedative use and fall-related outcomes in older adults.

## Conclusions

This study highlights patterns of fall-related emergency department visits among older adults and their association with demographic and clinical characteristics. Older age and cognitive conditions such as dementia were linked to a higher likelihood of falls, while differences were observed across sex and racial groups. An inverse association between sedative use and fall-related visits was observed; however, this finding should be interpreted with caution, as it may reflect underlying methodological factors, including confounding and exposure misclassification, rather than a true protective effect. These results underscore the complexity of evaluating fall risk in observational data and should not be used to inform changes in clinical practice. Future research should prioritize longitudinal study designs and incorporate detailed clinical measures, including functional status and medication patterns, to better clarify the relationship between sedative use and fall-related outcomes in this population.
